# Microvascular autologous submandibular gland transplantation in severe cases of keratoconjunctivitis sicca

**DOI:** 10.1186/s40902-015-0006-4

**Published:** 2015-02-05

**Authors:** Jia-Zeng Su, Zhi-Gang Cai, Guang-Yan Yu

**Affiliations:** Department of Oral and Maxillofacial Surgery, Peking University School and Hospital of Stomatology, No. 22, South Avenue Zhongguancun, Haidian District, Beijing, 100081 P.R. China

**Keywords:** Obstructive sialadenitis, Keratoconjunctivitis sicca, Submandibular gland transplantation

## Abstract

Dry eye syndrome is a relatively common disease of the tears and ocular surfaces that results in discomfort, visual disturbance, and tear film instability with possible damage to the ocular surfaces. Microvascular submandibular gland (SMG) transfer offers a surgical alternative for a permanent autologous substitution of tears using the basal secretion of a transplanted SMG. Long-term follow-up reveals that this technique is a lasting and effective solution for patients with severe dry eye syndrome. The uncomfortable symptoms were relieved, and the frequency of use of pharmaceutical tear substitutes was reduced. Objective examination showed significant improvement in tear film and some features of ocular surface such as breakup time of tear film and corneal staining. Patients may suffer from obstruction of Wharton's duct or epiphora after surgery. Activation of secretion-related receptors could improve the early hypofunction of the denervated SMG and prevent the duct obstruction. Reduction surgery, partial SMG transplantation, uses of atropine gel or Botulinum toxin A could be the choices of treatment for epiphora.

## Introduction

Keratoconjunctivitis sicca (KCS), or dry eye syndrom, is a multifactorial disease of the tears and ocular surfaces that results in discomfort, visual disturbance, and tear film instability with possible damage to the ocular surfaces [1]. The current options for treatment include artificial tear substitutes, cyclosporine eye drops, and occlusion of tear drainage [2,3]. However, these treatments have only satisfactory results in mild cases. For severe cases of KCS, none of the routine methods of symptom management are satisfactory [4].

Microvascular submandibular gland (SMG) transfer with implantation of Wharton’s duct into the upper conjunctival fornix offers a surgical alternative for a permanent autologous substitution of tears using the basal secretion of a transplanted and revascularized submandibular gland. This procedure was first described by Murube-Del-Castillo in 1986 [5]. His surgical attempt was followed by MacLeod [6], Kumar [7], Geerling [4], Sieg [8], Jia [9], Yu [10], and Paniello [11]. The reports from these groups confirmed that the secretions from all viable transplanted SMGs maintained stable function in the long term and had been effective for severe cases of KCS [12-17].

## Review

### Preoperative evaluation

Before surgery, a detailed medical history questionnaire regarding etiology, course and past treatment was taken to assess the dry eye symptoms subjectively. Detail and objective assessments, including ophthalmologic evaluation, oral and maxillofacial evaluation and scintigraphy with ^99m^Tc-pertechnetate were performed. The ophthalmologic evaluation included best-corrected visual acuity, Schirmer test, break-up time (BUT), and fluorescence staining of the cornea and conjunctiva. Oral and maxillofacial evaluation included wetting of the oral mucosa, saliva pooling at the floor of the mouth, salivary secretion at the ductal orifices of parotid and submandibular glands. Scientigraphy with ^99m^Tc-pertechnetate was carried out to test the secretion function of submandibular and parotid glands. Generally speaking, the SMG with a relative hypofunction was the first choice of donor gland [10,18,19].

The indications for the surgical procedure were that the patients had persisting pronounced symptoms of dry eye, that where other previous ophthalmologic treatments had failed, and that in the ophthalmologic evaluation, Schirmer test showed a value of <2 mm, the BUT a value of <5 sec, and that the cornea showed positive fluorescence staining. Contraindications were Sjőgren’s syndrome, obvious symptoms of xerostomia or that the whole saliva flow rate was <0.3 g/min, and that scintigraphy showed a severe dysfunction of multiple major salivary glands [18].

### Surgical procedure

**Figure 1:**
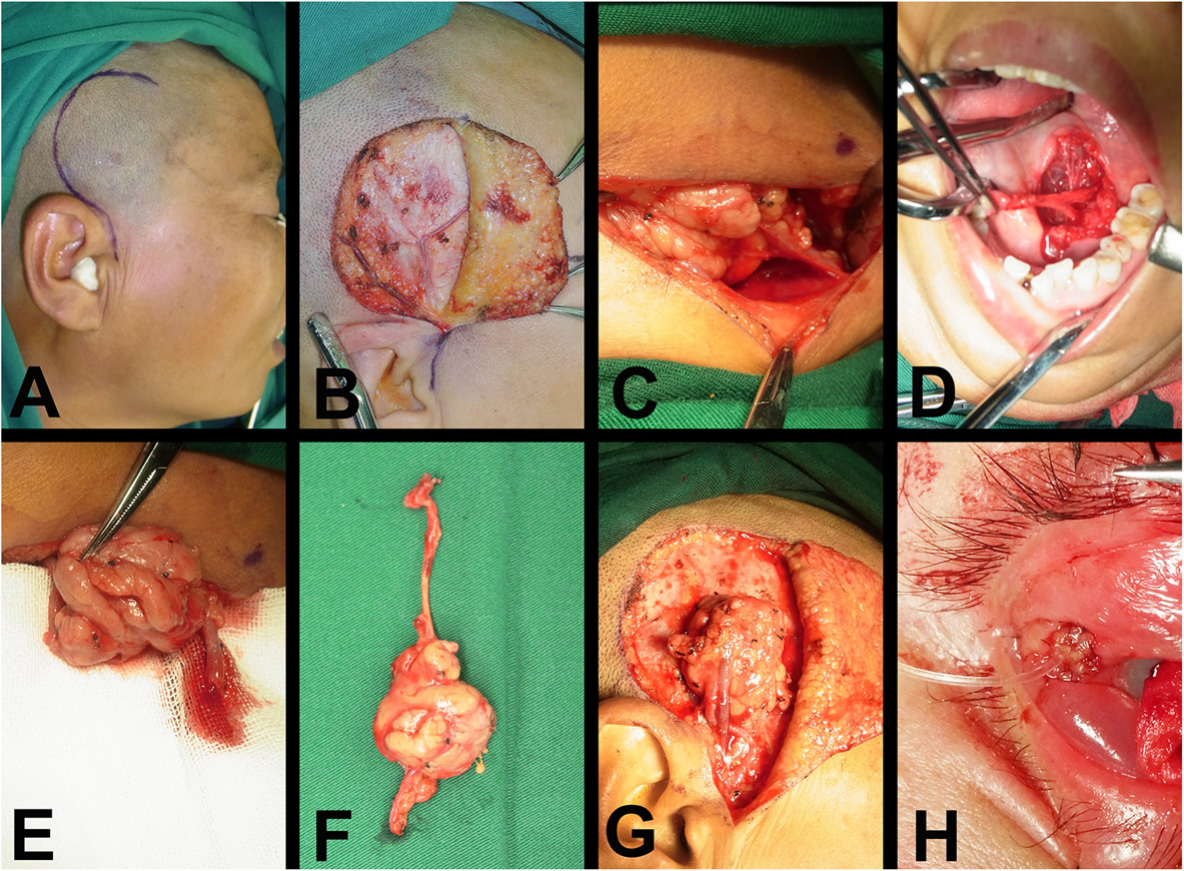
**Surgical procedures of autologous microvascular submandibular gland transplantation.** **(A)** Incision in the temporal region. **(B)** Dissection of the superficial temporal vessels. **(C)** Dissection of the facial artery and its accompanying vein. **(D)** Dissection of the Wharton’s duct and a cuff of mucosa around the opening. **(E)** Bleeding from the anterior facial vein which attached to the submandibular gland was observed to evaluate the state of venous drainage from the gland. **(F)** The submandibular gland was removed together with the facial vessels and Wharton’s duct. **(G)** Anastomosis of the vessels. **(H)** A nylon tube was inserted into the duct.

Under general anaesthesia, a curved incision was made in the temporal region (Figure 1A). An enlarged, caudally based temporal flap was retracted from the layer superficial to the superficial temporal vessels. Then the superficial vessels were carefully dissected (Figure 1B).

The submandibular gland, including Wharton’s duct and its related blood vessels, was harvested from the submandibular triangle through a conventional cervical approach. The dissection of the submandibular gland was taken using an extra-capsular approach to preserve the structures of the gland. The lower part of anterior facial vein, the proximal part of facial artery and its accompanying vein were carefully dissected and protected (Figure 1C). The chorda tympani supplying the submandibular gland was severed, whereas the lingual nerve was carefully protected.

The second part of the operation was performed intraorally. A blunt probe was inserted into the duct through its orifice to label Wharton’s duct. The mucosa was incised between the floor of the mouth and the base of the tongue, with a 5 cm length incision. A cuff of mucosa was left intact around the duct opening (Figure 1D). The duct was dissected and the lingual nerve was protected. The salivary duct of the sublingual gland which joins Wharton’s duct was cut and ligated. The entire Wharton’s duct was pulled out through the cervical submandibular access. Now the submandibular gland was released. Only the facial artery and lower part of anterior facial vein were left, connecting the submandibular gland to the body. The anterior facial vein was cut near the point joining itself with the common facial vein. Bleeding from the anterior facial vein which attached to the submandibular gland was observed to evaluate the state of venous drainage from the gland (Figure 1E). The proximal part of facial artery and its accompanying vein were cut at the roots of their branches from the external carotid artery. The submandibular gland was removed together with the facial vessels and Wharton’s duct (Figure 1F).

Heparinized saline was irrigated into the facial artery. The seepage of the solution from the anterior facial veins, accompanying vein of facial artery and the vein close to the duct was observed. The vein with most seepage of solution was taken as the first choice for anastomosis.

Afterwards, the free submandibular gland was transferred to the temporal region. The superficial temporal vessels were cut and prepared for anastomosis. The superficial temporal artery was anastomosed with the facial artery by means of a 8-0 suture. The superficial temporal vein was anastomosed with anterior facial vein or the accompanying vein of the facial artery (Figure 1G).

Wharton’s duct was passed through a tunnel prepared subcutaneously to the upper lateral conjunctival fornix by blunt dissection. The distal end of Wharton’s duct was sutured to form an opening in the upper lateral conjunctival fold. A nylon tube was inserted into the duct and fixed on the skin of the lateral wall of the orbit (Figure 1H) [10,18,19].

### Postoperative function regularity and ophthalmologic assessment

In the patients with successful transplantation, the secretion of the transplanted glands underwent a regular change after transplantation. It could be divided into four stages: transient hypofunction period (1-2 days after operation), temporary epiphora period (3-6 days after operation), latent period (1 week-3 months after operation) and function recovery and stable period (more than 3 months after operation) [10,20].

In the first 1-2 days after operation, very little secretion appeared. Then hypersecretion occurred in the “temporary epiphora period” (a mean value of 25 mm with Schirmer test). Secretion declined gradually over the next 3 months in “latent period” (mean value of Schirmer test, 5 mm). At last the secretion rate picked up again and remained stable after 3 months (mean value of Schirmer test, 17 mm) [10,20].

Postoperative ^99m^Tc-pertechnetate examination showed that the secretory function from all viable transplanted SMGs maintained stable function in the long term [21]. The lubricant effect of saliva appeared satisfactory with disappearance or relief of symptoms resulting from dry eye. The patients were able to eventually stop the application of artificial tear substitutes. Objective examination showed significant improvement in tear film and some features of ocular surface such as break-up time of tear film and corneal staining [10,13,15].

### Postoperative complications

#### Duct obstruction

The nylon tube inserted into the duct was kept for about 10 days, to prevent scar formation between the orifice of the duct and the conjunctiva and stricture of the duct opening. In some cases in which the orifices of the left and right Wharton’s ducts were very close to each other, harvesting of the mucosal cuff was abandoned in order to avoid injury to the contralateral ductal orifice. In these circumstances, Wharton’s duct had a naked end, and the ductal wall was directly sutured to the palpebral conjunctiva. For those patients a polyethylene tube would be intubated and left in the Wharton’s duct for more than 1 month.

The transplanted gland is completely disconnected from normal nerve supply during surgery. As a result, the gland is hyposecretory for about 3 months after transplantation. During this latent period, transplanted SMGs are at a high risk of duct obstruction, a complication that results in insufficient ocular lubrication of the treated eyes, greatly impairs the effect of transplantation, and may even lead to treatment failure. The incidence of this complication was about 9.3% in the early phase (0–3 months) [6,10,22]. The chronic inflammation secondary to ductal obstruction (obstructive sialadenitis) of the transplanted SMG showed very low secretion and recurrent swelling fo the gland. Viscous secretions were expressed upon milking the swollen transplanted glands [22].

Promotion of secretion from the transplanted SMG during the latent period is the main point for prevention of ductal obstruction. Secretions from normal SMGs are primarily evoked by the action of acetylcholine on muscarinic receptors and adrenergic agonists on adrenoceptors [23]. In our systemic studies on secretion of SMG, a functional expression of transient receptor potential vanilloid subtype 1 (TRPV1) was found in human and rabbit SMGs [24,25]. In the rabbit models of experimental transplanted SMG, carbachol (an agonist of muscarinic receptors) and capsaicin (an agonist of TRPV1) improved secretory function of transplanted SMG in the early stage after transplantation [25,26].

Clinically we found that combined administration of capsaicin and carbachol during the latent period could significantly increase the secretion of transplanted SMG and decrease the incidence of ductal obstruction [27]. As for the patients with duct obstruction of the transplanted SMG, reconstruction of the duct with venous grat or recontouring of the duct orifice would be the choice of treatment [10].

#### Epiphora

Secretion of the transplanted SMG is much more than that of lacrimal glands. Epiphora may occur in more than 40% of the patients with total SMG transplantation, especially in those with ample SMGs and normal function before surgery [10,21]. Severe epiphora may lead to social embarrassment and blurred vision. This probem is usually provoked by high room temperature or physical activity [4,28], It is usually treated by gland reduction surgery, that is, removing approximately one third to half of the transplanted SMG to decrease the glandular secretion. However, the patients should undergo additional surgery after transplantation. According to our experience, transplantation of partial SMG can reduce the incidence of severe postoperative epiphora for the patients with ample SMGs and normal function [12]. For the patients with mild or moderate epiphora, satisfactory treatment effect of topical application of atropine gel could be achieved [25]. While reduction surgery is used for the patients with severe epiphora. Botulinum toxin A is also used for control of over-secretion of the transplanted SMG [29,30].

## Conclusion

Microvascular SMG transfer is effective for the severe cases of KCS. A comprehensive evaluation on the eyes and gland is needed before surgery. The Wharton’s duct and the related blood vessels of SMG should be harvested intactly in the surgery. Venous drainage from the gland should be evaluated to choice the proper vein for anastomosis. Patients may suffer from obstruction of Wharton's duct or epiphora after surgery. Activation of secretion-related receptors could improve the early hypofunction of the denervated SMG and prevent the duct obstruction. Reduction surgery, partial SMG transplantation, uses of atropine gel or Botulinum toxin A could be the choices of treatment for epiphora.
